# Dosimetric impact of tracheostomy devices in head and neck cancer patients

**DOI:** 10.1002/acm2.12862

**Published:** 2020-05-06

**Authors:** Justin Lee, Sherif Ramadan, Anthony Kim, Yasir Alayed, Ananth Ravi

**Affiliations:** ^1^ Department of Radiation Oncology Odette Cancer Centre Sunnybrook Health Sciences Centre Toronto ON Canada; ^2^ Department of Radiation Oncology Juravinski Cancer Centre Hamilton ON Canada; ^3^ Department of Radiation Oncology University of Toronto Toronto ON Canada; ^4^ Department of Health Sciences McMaster University Hamilton ON Canada; ^5^ Department of Medical Physics Odette Cancer Centre Sunnybrook Health Sciences Center Toronto ON Canada

**Keywords:** head and neck cancer, optically stimulated luminescent dosimetry, radiation therapy, tracheostomy devices

## Abstract

**Introduction:**

The tracheostomy site and adjacent skin is at risk for recurrence in head/neck squamous cell cancer patients. The tracheostomy tube is an *in situ* device located directly over the tracheostomy site and may have clinical implications on the radiation dose delivered to the peristomal region. This study aimed to investigate this effect by comparing the prescribed treatment planning dose with the actual dose *in vivo* to the peristomal clinical target region. A retrospective, dosimetric study was performed with approval of the institutional research ethics board.

**Methods:**

Fifteen patients who had received high‐dose radiotherapy to the tracheostomy region with *in vivo* dose measurements were included. The radiation dose at the skin surface underneath the tracheostomy device was measured using an optically stimulated luminescent dosimeter (OSLD) and was compared with the prescribed dose from the radiation planning system. The effect of the tracheostomy flange and/or soft tissue equivalent bolus on the peristomal dose was calculated.

**Results and discussion:**

Patients with tracheostomy equipment *in situ* were found to have a 3.7% difference between their prescribed and actual dose. With a tissue equivalent bolus there was a 2.0% difference between predicted and actual. The mean prescribed single fraction dose (mean = 191.8 cGy, SD = 40.18) and OSLD measured dose (mean = 194.02 cGy, SD = 44.3) were found to have no significant difference. However, with the flange excluded from the planning simulation (density = air) target skin dose deviated from predicted by an average of 55.3% (range = 12.4–72.9, SD = 22.5) and volume coverage was not achieved.

**Conclusion:**

In summary, the tracheostomy flange acts like bolus with a twofold increase in the skin surface dose. Changes in the peristomal apparatus from simulation to treatment needs to be considered to ensure that the simulated dose and coverage is achieved.

## 1 INTRODUCTION

Radiotherapy is the primary treatment for many head and neck cancer patients and plays an important role in the postoperative setting for patients with locally advanced disease. When locally advanced tumors cause dyspnea, orthopnea, and stridor, patients may undergo an emergency tracheostomy procedure to protect the airway. In addition, tracheostomy is required following total laryngectomy and other radical surgeries to help manage secretions. This is clinically important as it can affect the ability to effectively deliver radiation therapy to this region. Rates of peristomal recurrence have been described between 1 and 11% and are associated with significant morbidity and mortality.^1^, ^2^, ^3^ Accurate dose delivery to the peristomal region is a key factor in reducing peristomal recurrence.^4^, ^5^ It is generally recommended that clinical target volumes (CTVs) include the stoma site and adjacent skin as these areas are at risk for locoregional recurrence in patients who had preoperative or intraoperative tracheostomy.^6^, ^7^

Although modern treatment planning systems (TPS) are reliably accurate for regions located beyond the depth of maximum dose, there remains an element of dosimetric uncertainty in the surface and build‐up regions.^8^ Linear accelerators emit significant levels of electron contamination (EC) that are difficult to model in a TPS that computes dose based upon kernel superposition methods. A common way to compensate for the EC problem is to empirically model the EC effect and superimpose it on the kernel superposition dose calculation. Although this empirical fit technique does improve the modeling of the surface/build‐up region considerably, accurate dosimetry is still a challenge for complex beam arrangements such as those seen in intensity‐modulated radiation therapy (IMRT).^9^ One indication of the challenges faced when modeling the build‐up region is demonstrated in AAPM’s Task Group 53 report on commissioning and quality assurance of TPS.^10^ In this report an example recommendation for build‐up region dose accuracy is stated as 20% for square, rectangular, and asymmetric fields. Modern TPS can typically achieve accuracies of better than ±10% for the surface region (0–0.5 cm depth), and ±5% in the build‐up region (0.5 cm to depth at maximum dose) for simple square fields.^11^, ^12^, ^13^

The issues around dose uncertainty in superficial regions is of particular relevance for head and neck cancer, where the planning target volume (PTV) often encroaches upon the patient surface.^14^ In the present IMRT application, it is desired to confirm that the dose received by peristomal tissue lying beneath the plastic/silicone components of a tracheostomy flange is at the desired level. Since this peristomal region is in the surface/build‐up region, there is an inherent uncertainty to the dose planned by the TPS. Chung et al^15^ reported a phantom study that simulated head and neck IMRT treatments for shallow (0.5 cm depth) and deep (6 cm depth) targets. Using Pinnacle 3 as the TPS and radio chromic film as the dosimeter, there was a 5.6% and 6.5% agreement for surface dose for the shallow and deep targets, respectively.

Due to the uncertainties in TPS predictions for dose in the surface/build‐up regions, *in vivo* dosimetry is occasionally required. This technique allows for direct measurements of the dose to ensure that the patient is exposed by the appropriate amount for the region of interest. Traditionally, TLDs, diodes, or metal oxide semiconductor field effect transistors (MOSFETs) have been used for *in vivo* dosimetry. Recently, dosimeters based upon optically stimulated luminescence (OSL) have been proven to be useful and increasingly popular.^16^, ^17^ The objective of taking direct OSL measurements of peri‐stomatic tissue is to confirm that the bolus effect of the tracheostomy equipment in the peristomal area is adequately modeled by the Pinnacle^3^ TPS.

To our knowledge the dosimetric effect of the actual tracheostomy tube and flange *in situ* has not been previously described. Therefore, a dosimetric study was performed to evaluate the impact of the tracheostomy hardware on the measured dose delivered to patients and the predicted dose calculated by the TPS.

## 2 MATERIALS AND METHODS

A retrospective, dosimetric study to assess the impact of tracheostomy hardware was performed with approval of the institutional research ethics board. All head and neck cancer patients were identified from a retrospective database and included in the study if they met several criteria. These criteria included patients who: had tracheostomy, received radiotherapy and had a physical OSL dosimeter (OSLD) measurement of the dose at the stoma site between 2013 and 2017. The dosimeter location was known to be a predetermined region associated with the highest prescribed dose from the planning distribution. This is an institutional policy that is followed for all patients.

### 2.A Radiation planning and treatment

Head and neck contouring was completed by the attending radiation oncologist based on the institutional standard agreed upon for contouring of organs at risk and target volumes.^18^, ^19^ The tracheostomy site and surrounding skin were considered to be a region at risk of microscopic disease and a CTV was contoured with a PTV margin of 5 mm. The prescribed doses were determined based on institutional practice and provincial guidelines.^19^ If macroscopic disease was present, the prescribed dose was 70 Gy. However, the range of prescribed doses in this study reflects various clinical factors such as disease site, stage, high‐priority dose‐limiting structures, prior surgery, or presence of macroscopic disease. Intensity‐modulated radiation treatment (IMRT) planning for head and neck cancer patients was performed using a Philips Pinnacle^3^ TPS version 9.2 (Philips Medical Systems, Andover, MA) for all patients. Plans typically employed a six or seven co‐planar beam arrangement with additional noncoplanar beams if required. Patients were aligned in the supine position and immobilized with a thermoplastic mask. No treatment plans using electrons, orthovoltage tomotherapy, or VOLUMETRIC arc therapy (VMAT) were included in this study. The IMRT plans were optimized so that at least ninety five percent of tracheostomy site PTV received the prescribed dose, for example, V56 Gy > 95%. The radiation treatment plans for all patients were copied to a research database for review and dosimetric analysis.

### 2.B Optically stimulated luminescence (OSL) measurements *in vivo*

For *in vivo* dosimetry, a commercial OSL system was used consisting of the InLight microStar reader (Landauer, Glenwood, IL) with Landauer nanoDot dosimeters. These devices were prescreened by the manufacturer for accuracy. This system can be used to measure dose at or near the skin surface.^20^, ^21^ The OSL sensitive material is aluminum oxide with carbon impurities (Al_2_O_3_:C) encapsulated in 0.2 mm thick, 5 mm diameter discs. This sensitive material is enveloped by a light‐tight plastic casing that measures 10 × 10 × 2 mm. In the first year of using OSLDs at our institution, monthly quality control tests determined that the OSL system is accurate within ±3% for therapeutic doses (approximately 10–300 cGy/fraction) These results are in line with the manufacturer's specifications and prior data.^20^, ^21^ Quality control tests were then performed on an ongoing basis to ensure that the OSLD measurements stayed within this accuracy range.

### 2.C Quality assurance/verification

For any high dose head and neck radiotherapy plan, the treating radiation oncologist may request OSLD measurement for verification of the delivered dose relative to the planned dose. The institutional policy was that the measurement would not result in any treatment changes unless a discrepancy of >5% was detected and felt to be clinically significant. An OSLD measurement was performed for one of the fractions during the treatment course for each individual patient involved in this study. Once the patient was set up on the treatment couch, an OSLD was placed on the skin directly adjacent to the stoma. This was a predetermined region associated with the highest prescribed dose from the planning distribution. The tracheostomy flange was located directly over top of the OSLD and centered over the stoma as per the usual clinical practice. The OSLDs were left on during cone‐beam CT imaging for positioning and verification. This was done for practical purposes, since patients were treated in an immobilizing thermoplastic mask. This made it unfeasible to position the patient with CBCT and then place the OSLD under the mask and other apparatus. It was determined that the CBCT dose only added between 0.2 and 0.6 cGy of dose reading (uncorrected for the kV response of the OSLDs) to the OSLD during a phantom study. This was determined with OSLDs that were taped to an anthropomorphic head‐and‐neck phantom (Rando® phantom, The Phantom Laboratory, Salem, NY). These phantoms were then CBCT‐scanned using the clinical H&N settings. The imaging dose was a small, negligible percentage as compared to the typical 200 cGy dose per fraction delivered in a head and neck radiation treatment plan.

**Fig. 1:**
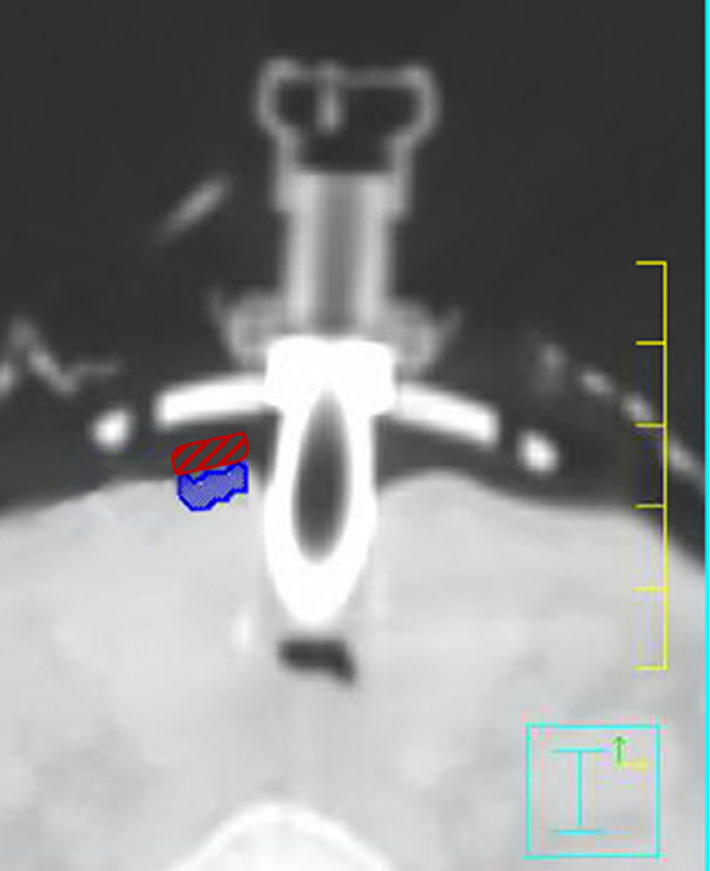
Screenshot of the peristomal target region (blue) underneath the tracheostomy flange (bright white), which demonstrates where dose calculations were made. The red contour approximates the placement of the optically stimulated luminescent during treatment just superior to the peristomal region.

After irradiation, the OSLDs were read out by the microStar reader after at least 10 min had elapsed.^22^ Each OSLD was read out three times, and the results were averaged and then reported. These results were compared to the OSLD dose as predicted by the TPS in the patient treatment plan. A contour was then drawn to approximate the OSLD in the location that the dosimeter was placed during the treatment fraction as seen in Fig. 1. The mean dose to this OSLD was reported and compared to the OSLD reading.

### 2.D Measurement of tracheostomy material density

**Fig. 2:**
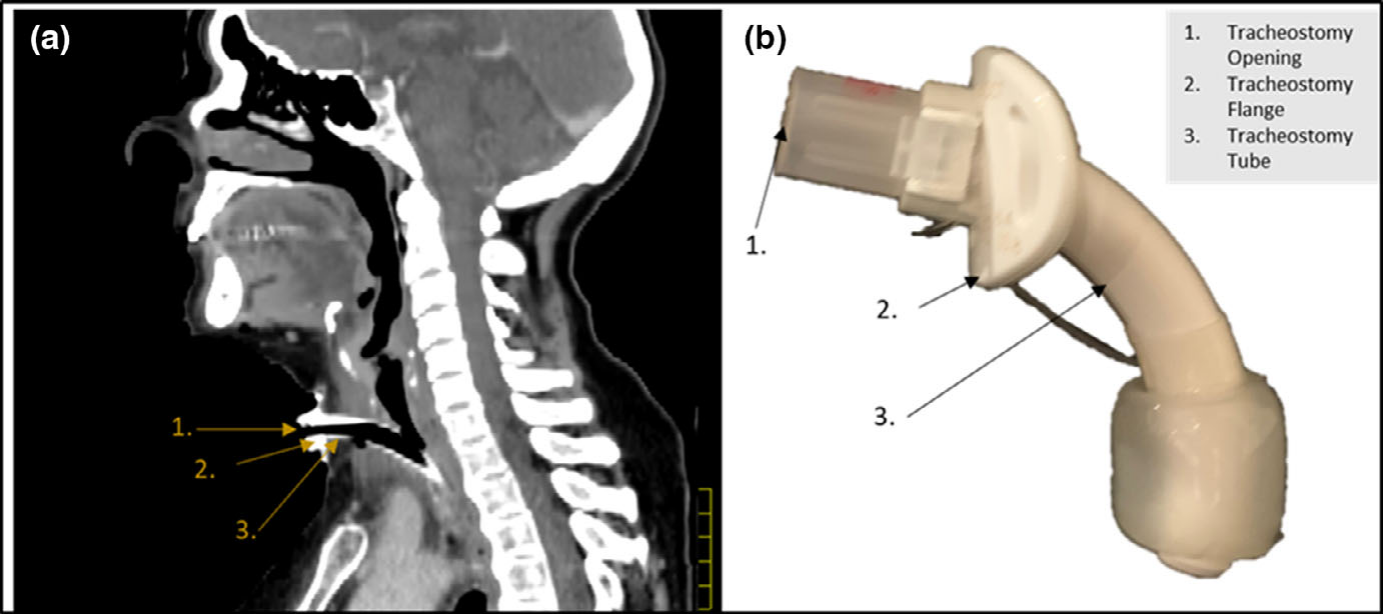
(a) A representative Sagittal view of the tracheostomy hardware placement can be seen. (b) A physical representation of the device including the opening, flange, and tube are displayed.

The density of the tracheostomy hardware was calculated using CT images. This result was then compared to physical measurements of the device’s density. This was done in order to ensure that the tracheostomy tube and flange were not composed of any high atomic number elements that would erroneously increase the calculated density on CT images,

CT images of patients with a tracheostomy tube and flange were acquired. These scans were obtained using a Philips Brilliance Big Bore CT scanner (Philips Healthcare, Best, The Netherlands). The images were acquired at 1.2 mm × 1.2 mm axial pixel pitch with a 1.5 mm voxel thickness using 120 kVp. The CT images were used to delineate the tracheostomy tube and the flange. The mean density of the flange volume was then calculated using Pinnacle^3^ TPS. The physical measurements of the density involved careful determination of flange volume using the Archimedes' principle of displacement.^23^ This was followed by an accurate measurement of the objects mass with a calibrated scale. A Sagittal view and physical representation of the tracheostomy placement and hardware can be seen in Fig. 2.

### 2.E Dosimetric plan evaluation

In order to evaluate the tracheostomy tube and flange’s impact on the dose to the adjacent target region, a retrospective dosimetric analysis was performed. The OSLD location was contoured using a 5 × 5 × 1 mm volume at the skin surface directly adjacent to the stoma site. This was done to estimate a CTV and this region was defined as the *peristomal volume*. The tracheostomy tube and flange were then contoured using a fine 1 mm brush on lung density CT window (W:1601, L:‐300) and is referred to as the *trach contour.* The mean dose to this OSLD volume was measured from the published patient treatment plan.

The number of monitor units was held constant for each patient’s treatment plan and then a smaller dose grid with a resolution of 0.1 mm was used for further analysis. To estimate the dose to the *peristomal volume* without the tracheostomy equipment, the density of the *trach contour* was set to zero and the dose was recomputed.

**Fig. 3:**
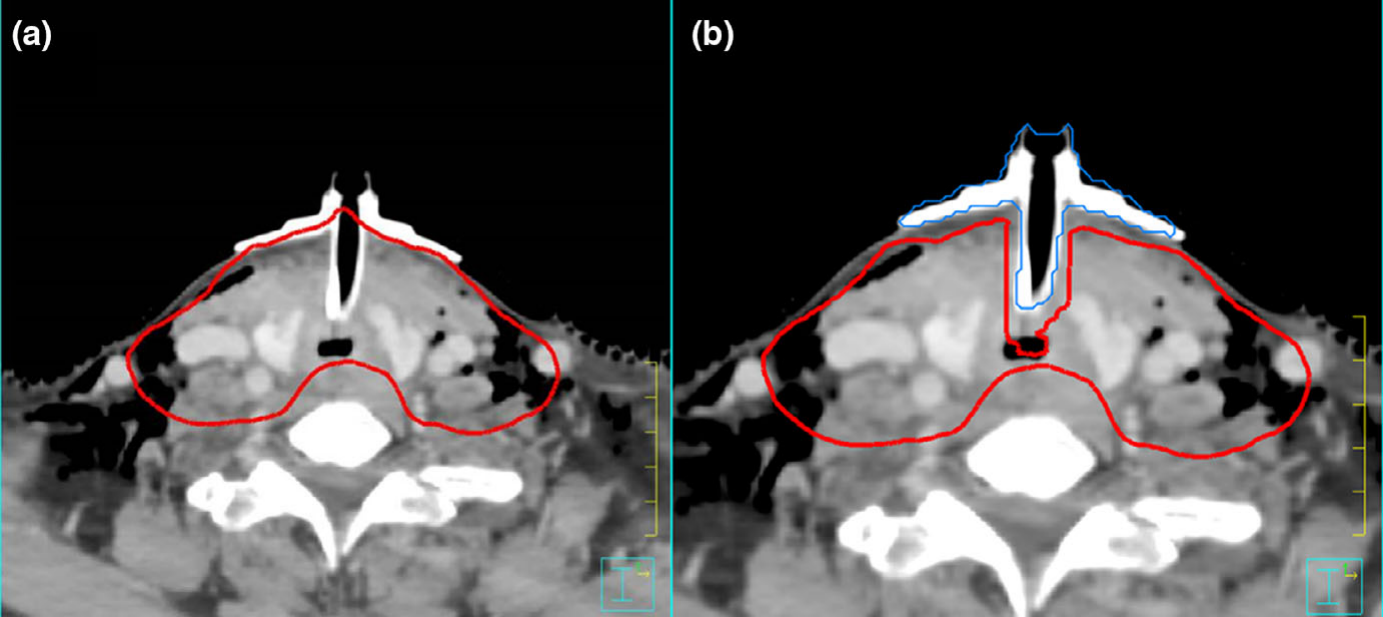
(a) Screenshot of a Tracheostomy flange and tube with the 95% isodose line(red). (b) With the tracheostomy flange and tube (blue) density set to air equivalent the treatment isodose line (red) shifts slightly below the skin surface. This causes a clinically significant impact on the treatment region.

For cases where additional bolus material was applied overtop of the tracheostomy flange, this additional material was contoured. The treatment plans were then evaluated again with normal density and then a second time with the bolus density set to zero using fixed monitor units for each plan. A screenshot of the tracheostomy tube, flange, 95% isodose line, and the effect that zeroing the trach contour has on the isodose line can be seen in Fig. 3.

### 2.F Statistical methods

The patient, tumor, and treatment characteristics (n = 15) were analyzed and summarized using descriptive statistics. The difference between the prescribed dose from the radiation plan and the measured OSLD dose was calculated as an absolute value (cGy) and as a percentage difference to normalize for the variation in the absolute prescribed doses. The mean of the differences was calculated along with the standard deviation of the differences. For each patient, a paired t‐test was carried out to assess the mean difference between the OSLD measurement and the radiation dose predicted by the planning system at the peristomal region.

## 3 RESULTS

This single institution study identified 15 patients with biopsy proven head and neck cancer. These patients had tracheostomy prior to radiation and at least one measurement of the dose received using an OSLD. A complete set of patient, tumor, and characteristic information can be seen in Table 1. Density of the tracheostomy flange was measured directly to be 1.189 ± 0.2 g/cm^3^ which was comparable to the predicted value from CT planning datasets, measured as 1.168 ± 0.2 g/cm^3^. Radiation plans consisted of IMRT technique with the prescribed dose to the tracheostomy site ranging from 50 to 70 Gy (median 70 Gy). Tables 2 and 3 outline the complete set of dose data for the 15 patients.

**Table 1:** Patient, tumor, and treatment characteristics.

Age	Range 40–90, Median 62
Sex	Male: 11 Female: 4
Primary tumor site	Larynx: 8 Oral cavity/Oropharynx: 4 Hypopharynx: 2 Thyroid :1
Pathology	Squamous cell carcinoma: 14 Anaplastic carcinoma (thyroid): 1
TNM Stage (*UICC 7th ed.)*	T4: 8 T3: 7 N0: 5 N1:2 N2: 5 Nx: 4 M0: 14 Stage III: 5 Stage IVA: 9
Radiation intent/indication	Adjuvant/postoperative: 9 Radical/curative (no surgery): 5 Palliative: 1
Planned radiation dose/prescription to tumor and nodes	<60 Gy: 1 60.0–69.9 Gy: 6 ≥70 Gy: 8
Planned dose to peristomal region	<50 Gy: 3 50–60 Gy: 6 >60 Gy: 6

**Table 2:** Peristomal dose in patients with tracheostomy equipment *In Situ.*

Patient number	Dose_OSLD_ (Gy)	Dose_Plan_ (Gy)	Dose_Plan, trach ρ = air_ (Gy)
1	50.96	51.20	20.65
2	64.68	62.74	19.59
3	58.09	56.67	16.87
4	50.29	54.27	14.70
5	28.30	26.83	15.91
6	25.32	24.91	8.41
7	31.68	33.43	11.24
8	66.00	62.04	17.42
9	51.48	53.99	18.69
10	66.36	64.52	14.89

Note

Dose_OSLD_, measured OSLD dose; Dose_Plan_, planned pinnacle dose; Dose_Plan, trach ρ = air_, Planned Pinnacle dose with trach density set to air equivalent. Tracheostomy equipment consists of the tracheostomy tube and flange and is referred to as “trach”.

**Table 3:** Peristomal dose in patients with bolus and/or tracheostomy equipment.

Patient number	Dose_OSLD_ (Gy)	Dose_Plan_ (Gy)	Dose_Plan, Trach ρ = air Bolus ρ=actual_ (Gy)	Dose_Plan, Trach + Bolus ρ = air_ (Gy)
11	73.26	71.50	46.85	28.80
12	65.67	64.31	56.35	21.52
13	71.28	68.76	59.67	26.29
14 (no applicator)	58.59	58.51	N/A	30.65
15 (no applicator)	50.20	51.14	N/A	27.67

Note

Patients 11–15 all had 1‐cm‐thick tissue equivalent bolus placed over the tracheostomy site. Within this group, Patients 11–13 had both tracheostomy equipment and bolus over the tracheostomy site. Dose_OSLD_, Measured OSLD dose; Dose_Plan_, planned pinnacle dose; Dose_Plan, Trach ρ = air and Bolus ρ = actual_, Planned Pinnacle dose with bolus density not modified and only trach density set to air equivalent; Dose_Plan, Trach + Bolus ρ = air_, Planned Pinnacle dose with trach density and bolus density both set to air equivalent.

**Fig. 4:**
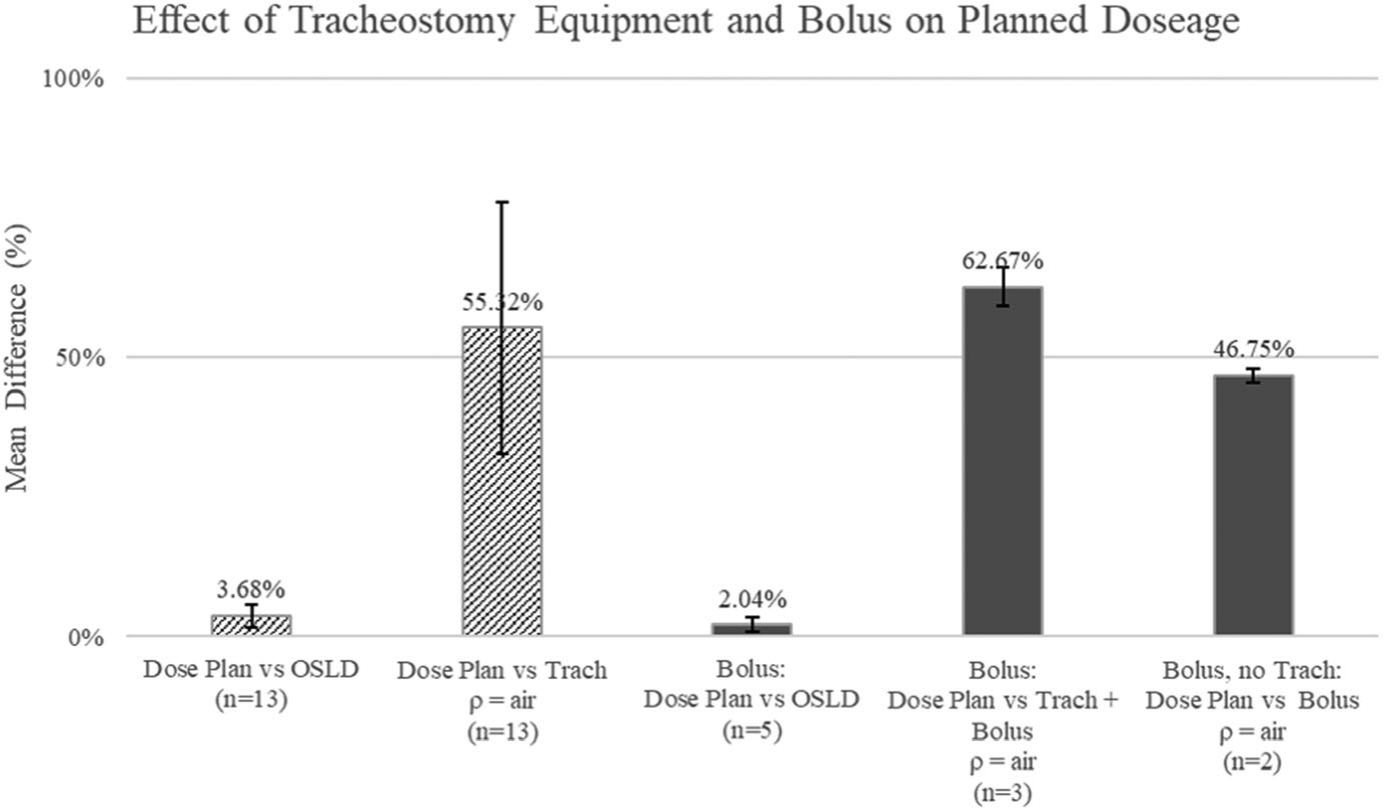
Mean difference between the measured, planned, and predicted dose plans for patients receiving treatment with/without bolus. Dose plan refers to the original dose calculated by the pinnacle plan. Originally there is minimal deviation between the planned dosage and the measured dosages from the optically stimulated luminescent dosimeter with or without a bolus. When the trach/bolus were set to air equivalent (ρ = air) a new predicted dose was calculated by the pinnacle software, this shows the effect that a bolus or tracheostomy equipment has on the planned pinnacle dosage. For the 13 patients that did not have a bolus, setting the Trach density to air equivalent changed the calculated plan by 55%. For the three patients with a Bolus, and the two patients with a bolus and no Trach, setting the Bolus/Trach density to air equivalent changed the plan by 62% and 46%, respectively.

In Fig. 4 the average percentage difference between the various measured and planned dosages can be seen. For patients with tracheostomy equipment in place the average percentage difference between prescribed and actual measured dose was 3.8% (SD 2.1). When tissue equivalent bolus material was used, with or without a tracheostomy tube and flange, the average difference between the predicted and actual doses was 2.0% (SD 1.3). For all patients, a paired t‐test was carried out to compare the prescribed dose to the OSLD measured dose in the peristomal region of interest. There was no significant difference between the prescribed dose (mean = 191.5 cGy, SD = 41.7) and the measured dose (mean 193.5 cGy, SD = 46.0); t(13) = 0.99, *P* = 0.34.

When the *trach contour* was excluded from the planning CT scan (density set to air equivalent) the target coverage and dose to the *peristomal volume* decreased significantly. The predicted mean dose was reduced by an average of 53.5% (SD 22.5) and therefore coverage of the peristomal target volume was inadequate.

For patients with bolus, the target dose and coverage also decreased when the bolus and applicator were excluded from the planning CT (density set to air equivalent). This was seen to be an average of 62.7% (SD 3.50) for patients with a bolus and an applicator and 46.8% (SD = 1.22%) for patients with a bolus but no applicator.

## 4 DISCUSSION

This study demonstrates that the tracheostomy tube and flange have a significant impact on head and neck radiation target coverage in the peristomal region. This was achieved through analysis of 15 separate patients. Patients 1–10 only had a tracheostomy device and did not have any bolus material. With the tracheostomy device density set to air equivalent a significant difference between the predicted plans can be seen. For patients 11–13 a bolus material was present in addition to the tracheostomy device. By comparing the dose changes with the tracheostomy device and bolus set to air equivalent we can directly observe the similar effect that these two materials have on the predicted dosage. Additionally, patients 14 and 15 further reinforce this conclusion, as even without a tracheostomy device the significant dose effect of the bolus can be seen. This dose effect is caused by how the radiotherapy beams interact with materials. Radiotherapy penetrates the surface layer it encounters and then irradiates deeper layers. Therefore, to treat superficial lesions a bolus material is commonly used to act as a layer of scattering material to replicate the skin surface. By setting the density of our tracheostomy applicator to zero we see a significant drop in the radiation dose delivered to the peristomal region. This directly demonstrates how the tracheostomy device is acting as a bolus material to cause an increase in the dose that the skin surface and subcutaneous tissues receive. To our knowledge, this is the first study to demonstrate the effect of tracheostomy equipment on radiation dose, using *in vivo* surface measurements to validate the predicted doses.

An important consideration for these findings is the accuracy of the OSLD measurements, and the differences between the measured and planned dosages. There are a couple of factors which can cause deviation between these values. The first major cause is related to the location of the dosimetry measurements. The dosimeters were placed on the skin directly adjacent to the stoma, within a region of high‐dose gradient. In this region there is the largest potential for deviation between the OSLD measurement and predicted dose value. Since there is a large gradient in this area, minor changes in positional accuracy will have large effects on the OSLD's measurement accuracy as compared to the predicted value. Second, the calculated TPS density for the applicator flap could potentially cause a deviation from the actual radiation dose delivered. However, the calculated density from the CT images of the applicator flap correlated with the measured physical density. This indicates that the applicator is constructed out of a polymer material with a low average atomic number; as such, the electron density used for the dose computation is accurate. Despite these sources of potential error, the difference between the planned and measured doses were on average <4%. This is within acceptable limits as described by evidence‐based treatment guidelines.^19^ Accurate dosimetry is important to ensure proper treatment delivery and can help to limit peristomal recurrence, particularly for head/neck squamous cell cancer patients.

As seen with the tracheostomy devices in this study, medical devices can have a significant effect on radiation treatment planning. The dosage effects of medical devices must be carefully accounted for to ensure target coverage and to avoid excessive toxicity.^24^, ^25^ An optimal radiotherapy plan needs to be able to effectively deliver the radiation dose to the targeted treatment area while minimizing dose delivery to adjacent structures. To achieve this goal the impact of any internal or external medical devices must be measured and accounted for in the radiation plan. The impact of dental implants and amalgam, intravenous ports, and breast and hip prosthesis has previously been described.^26^, ^27^, ^28^, ^29^ These devices may create dose inhomogeneity with the potential for increased toxicity or inadequate target coverage. Different medical devices can have unique impacts on the delivered radiation dosage and should be individually evaluated for their potential effect on the prescribed treatment plan.

This study has several important limitations. This was a retrospective study with variation in the clinical presentation, primary disease sites, and total prescribed dose, which reflects clinical practice. All patients included in the study had IMRT and therefore it is not possible to extrapolate these results to patients who may undergo other treatment techniques/modalities such as conventional radiotherapy, VMAT, electrons, or proton therapy. The OSLD measurements were taken on average once or twice per patient and it is possible that there may be differences in setup due to air gaps, device placement or inter/intra‐treatment motion through the full course of radiotherapy. The number of patients included in this study is low and reflects an uncommon but important clinical situation. Future studies are needed with a larger cohort of patients to allow for further statistical validation of these findings for specific clinical presentations and disease sites.

## 5 CONCLUSIONS

This is a retrospective, dosimetric study of 15 head and neck cancer patients who underwent high‐dose radiotherapy and had *in vivo* OSLD measurements at the peristomal region. The tracheostomy flange applicator was found to have a density similar to water equivalent bolus. Using OSLD measurements during treatments, the actual measured dose in the peristomal tissues was, on average, within 4% of the predicted dose from the radiation treatment plan. This was deemed to be clinically acceptable. Overall the tracheostomy flange causes a bolus effect, with a twofold increase in the skin surface dose. If there is no tracheostomy tube or flange in place, then a customized ring of bolus 1 cm thick, may be used to achieve similar dose coverage at the skin surface. Dose coverage of the tracheostomy site should be carefully evaluated for in each patient depending on the specific *in situ* device and institutional practice. Any changes that may occur in the peristomal device placement from the time of simulation to treatment completion could have a clinically significant impact on radiation dose and may require adaptive planning or other modifications.

## CONFLICT OF INTEREST

The authors do not have any conflicts of interest to declare.
